# Supplementary figures and images for: Disentangling conditional effects of multiple regime shifts on Atlantic cod productivity
Source: PLoS One. 2020 Nov 30;15(11):e0237414. doi: 10.1371/journal.pone.0237414 (PMC7703953; doi:10.1371/journal.pone.0237414)

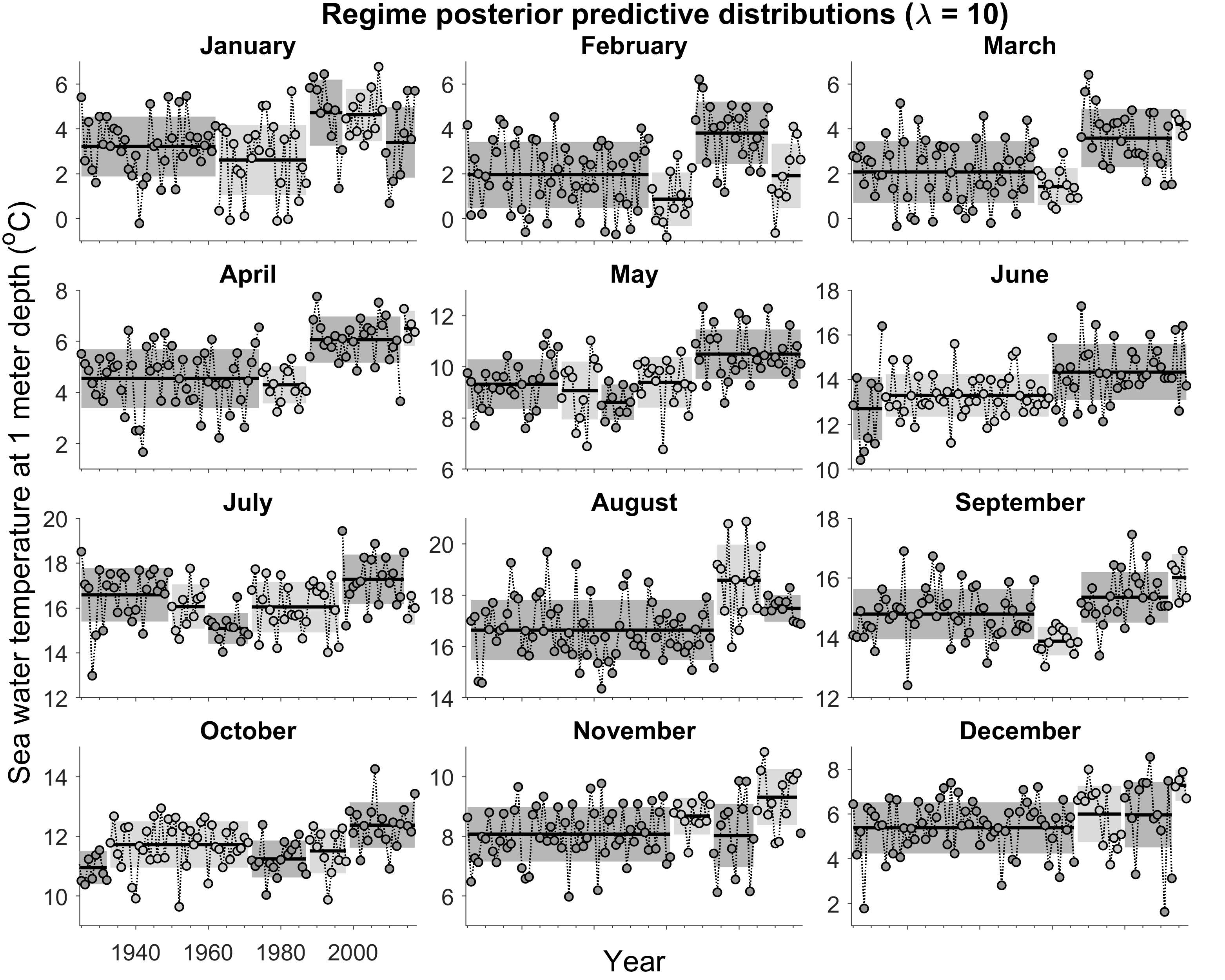

Supplement: S1 Fig — The shaded area represents the 68% central probability interval (CPI) of the posterior predictive distribution; thus, it includes uncertainty about the mean and the variance. Horizontal lines in each shaded region represents the mean. (PNG) [file pone.0237414.s001.png]

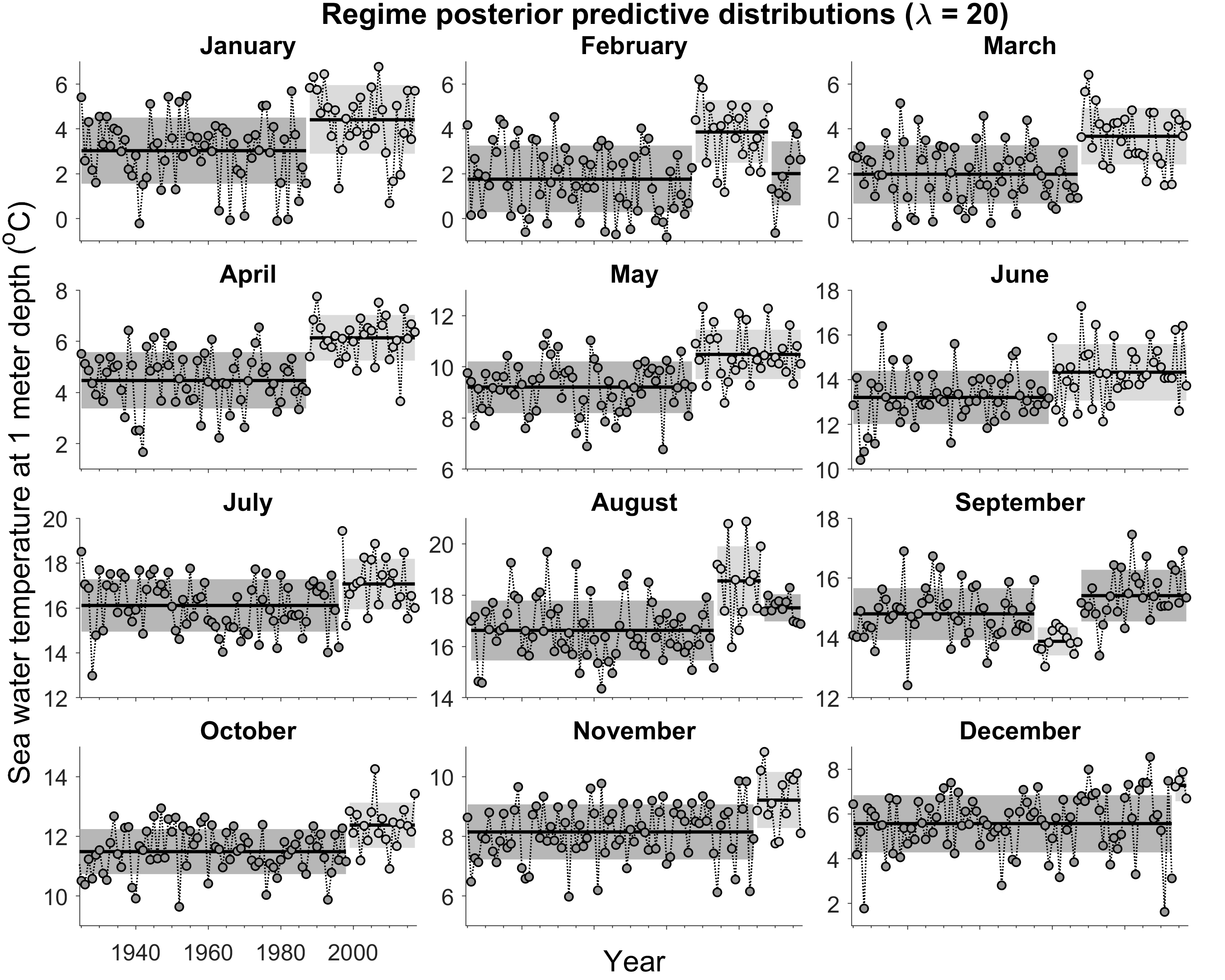

Supplement: S2 Fig — See the caption for Fig 1 or S1 Fig for explanations of the shaded regions of the data. (PNG) [file pone.0237414.s002.png]

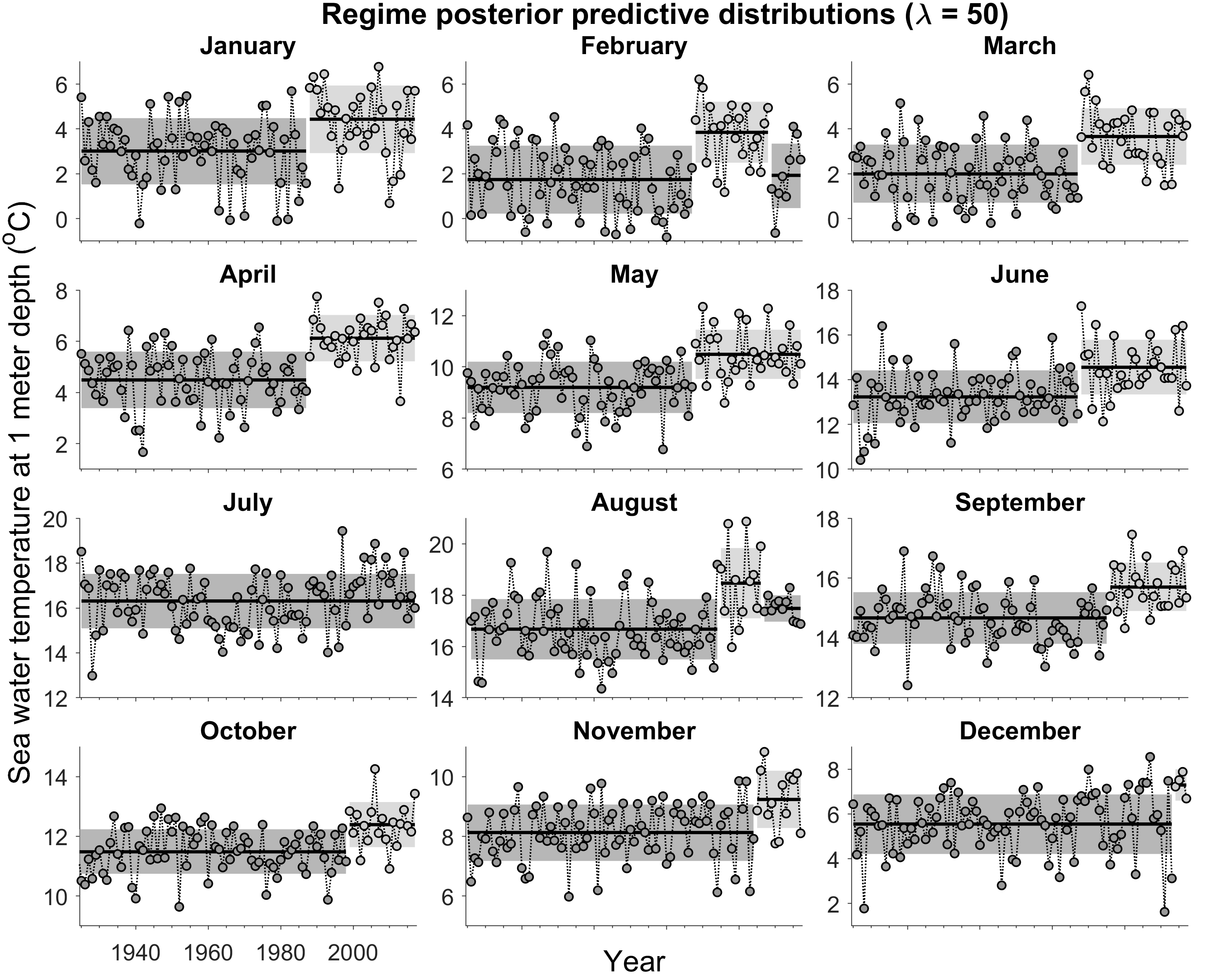

Supplement: S3 Fig — See the caption for Fig 1 or S1 Fig for explanations of the shaded regions of the data. (PNG) [file pone.0237414.s003.png]
